# Phase II trial of combination therapy of gemcitabine plus anti-angiogenic vaccination of elpamotide in patients with advanced or recurrent biliary tract cancer

**DOI:** 10.1007/s10637-014-0197-z

**Published:** 2014-12-13

**Authors:** Masato Matsuyama, H. Ishii, J. Furuse, S. Ohkawa, H. Maguchi, N. Mizuno, T. Yamaguchi, T. Ioka, T. Ajiki, M. Ikeda, K. Hakamada, M. Yamamoto, H. Yamaue, K. Eguchi, W. Ichikawa, M. Miyazaki, Y. Ohashi, Y. Sasaki

**Affiliations:** 1 Gastroenterological Medicine, Cancer Institute Hospital of Japanese Foundation for Cancer Research, 3-8-31 Ariake, Koto-ku, Tokyo, 135-8550 Japan; 2 Department of Medical Oncology, Kyorin University School of Medicine, Tokyo, Japan; 3 Division of Hepatobiliary and Pancreatic Oncology, Kanagawa Cancer Center Hospital, Kanagawa, Japan; 4 Center for Gastroenterology, Teine-Keijinkai Hospital, Hokkaido, Japan; 5 Department of Gastroenterology, Aichi Cancer Center Hospital, Aichi, Japan; 6 Department of Gastroenterology, Chiba Cancer Center, Chiba, Japan; 7 Hepatobiliary and Pancreatic Oncology, Osaka Medical Center for Cancer and Cardiovascular Diseases, Osaka, Japan; 8 Hepato-Biliary-Pancreatic Surgery, Kobe University Graduate School of Medicine, Hyogo, Japan; 9 Department of Hepatobiliary and Pancreatic Oncology, National Cancer Center Hospital East, Chiba, Japan; 10 Department of Surgery, Hirosaki University Graduate School of Medicine, Aomori, Japan; 11 Institute of Gastroenterology, Tokyo Women’s Medical University, Tokyo, Japan; 12 Second Department of Surgery, Wakayama Medical University, Wakayama, Japan; 13 Division of Medical Oncology, Teikyo University School of Medicine, Tokyo, Japan; 14 Division of Medical Oncology, Showa University School of Medicine, Tokyo, Japan; 15 Department of General Surgery, Chiba University Graduate School of Medicine, Chiba, Japan; 16 Department of Integrated Science and Engineering for Sustainable Society, Chuo University, Tokyo, Japan

**Keywords:** Biliary tract cancer, Immunotherapy, Cancer vaccines, Phase II clinical trial, VEGFR2

## Abstract

*Background* Elpamotide is an HLA-A*24:02-restricted epitope peptide of vascular endothelial growth factor receptor 2 (VEGFR-2) and induces cytotoxic T lymphocytes (CTLs) against VEGFR-2/KDR. Given the high expression of VEGFR-2 in biliary tract cancer, combination chemoimmunotherapy with elpamotide and gemcitabine holds promise as a new therapy. *Patients and Methods* Patients with unresectable advanced or recurrent biliary tract cancer were included in this single-arm phase II trial, with the primary endpoint of overall survival. Survival analysis was performed in comparison with historical control data. The patients concurrently received gemcitabine once a week for 3 weeks (the fourth week was skipped) and elpamotide once a week for 4 weeks. *Results* Fifty-five patients were registered, of which 54 received the regimen and were included in the full analysis set as well as the safety analysis set. Median survival was 10.1 months, which was longer than the historical control, and the 1-year survival rate was 44.4 %. Of these patients, injection site reactions were observed in 64.8 %, in whom median survival was significantly longer (14.8 months) compared to those with no injection site reactions (5.7 months). The response rate was 18.5 %, and all who responded exhibited injection site reactions. Serious adverse reactions were observed in five patients (9 %), and there were no treatment-related deaths. *Conclusion* Gemcitabine and elpamotide combination therapy was tolerable and had a moderate antitumor effect. For future development of therapies, it will be necessary to optimize the target population for which therapeutic effects could be expected.

## Introduction

In Japan, the incidence of biliary tract cancer (BTC) was ranked the sixth leading cause of cancer death in 2012. Although BTC is rare in Europe and America, it is highly prevalent in Japan, Chile, and East Asia [1, 2], presenting a serious health concern. The only hope for a complete cure is early-stage surgical resection. However, many BTC cases are unresectable due to locally advanced or distant metastasis. Moreover, recurrence after curative resection is not rare. Therefore, effective pharmacotherapies must be developed.

Vascular endothelial growth factor (VEGF)-A and its receptor, VEGF receptor (VEGFR), is highly expressed in many tumors including BTC [3]. VEGFR-2/KDR strongly promotes tumor angiogenesis, and active immunization against VEGFR-2/KDR has been reported to inhibit tumor growth and metastasis [4]. Thus, VEGFR-2/KDR holds hope as a target for tumor immunotherapy. Elpamotide, an HLA-A*24:02-restricted epitope peptide derived from VEGFR-2/KDR (KDR169), induces cytotoxic T lymphocytes (CTLs) that specifically recognize VEGFR-2/KDR169. These CTLs target tumor vascular endothelial cells that express KDR169-presenting HLA molecules, i.e., VEGFR-2/KDR expressing cells.

In this study, we assessed the efficacy and safety of combination immunotherapy with gemcitabine (Gem) and elpamotide in patients with BTC.

## Methods

### Study design

This multicenter, open-labeled, single-arm, phase II trial, which recruited patients via central registration, was conducted in accordance with the Declaration of Helsinki and the Standards for the Implementation of Clinical Trials on Pharmaceutical Products. The primary endpoint was overall survival, and secondary endpoints included progression-free survival and tumor regression. Sixteen facilities participated in this trial. This study was registered with UMIN, Clinical Trials Registry before the enrollment of the first subject (Registration number: UMIN000002500). Inclusion criteria of this trail were shown in Table 1.

**Table 1:** Criteria of this trial

Inclusion criteria
a) pathologically diagnosed adenocarcinoma or adenosquamous carcinoma with bile duct origin (extrahepatic bile duct, intrahepatic bile duct, gallbladder, or vater papilla)
b) unresectable or recurrent disease
c) HLA-A*24:02 positive
d) aged ≥20 years and <75 years
e) ECOG performance status of 0 or 1
f) expected to live for ≥3 months
g) adequate organ function meeting the following criteria: white blood cell count ≥3500/mm^3^ and ≤12,000/mm^3^, neutrophil count ≥2000/mm^3^, hemoglobin ≥9.0 g/dL, platelet count ≥100,000/mm^3^, total bilirubin ≤2.0 mg/dL, aspartate aminotransferase ≤150 IU/L, alanine aminotransferase ≤150 IU/L, and serum creatinine ≤1.5 mg/dL;
h) no previous history of chemotherapy, radiotherapy, or immunotherapy for BTC (eligible if adjuvant therapy with S-1 was performed ≥6 months before registration)
i) if underwent laparotomy, it was performed ≥2 weeks before registration
j) provision of written informed consent.
Criteria for discontinuation
a) when the primary disease observably worsened
b) when dose reduction of Gem was required for more than two stages
c) when adverse events made continuation difficult
d) when treatment was postponed for more than 28 days
e) when 1.5 years had passed from registration

### Study treatment

One course of elpamotide (4 weeks) consisted of a single weekly subcutaneous injection (2.0 mg/mL/body) on day 1, day 8, day 15, and day 22. One course of Gem (4 weeks) consisted of a single weekly mediation (1000 mg/m^2^/30 min) on day 1, day 8, and day 15 (day 22 was skipped). Criteria for discontinuation were shown in Table 1.

### Efficacy and safety

Restaging CT was performed every 6 weeks and evaluated according to RECIST criteria version 1.1. The final tumor regression effect was determined by consensus of the image evaluation committee. Overall survival was defined as time from the day of registration to the day of death from any cause or 1.5 years afterwards. Progression-free survival was counted from the day of registration to the day of progressive disease by clinical evaluation or imaging diagnosis, whichever was earlier.

Adverse events were evaluated at each hospital visit and graded according to the Common Toxicity Criteria version 3 (CTCAE v3). Adverse events which could not be ruled out as being related to the trial therapy were reported as adverse drug reactions (ADRs). For each adverse event, we documented the worst grade for each patient, and confirmed the incidence of each by grade.

### Exploratory assessment

Induction of VEGFR-2-specific CTLs and serum concentrations of VEGFR-2 were analyzed only in subjects who provided specific consent to receive these assessments at some of the participating medical institutions.

The induction of VEGFR-2-specific CTLs was evaluated by an enzyme-linked immunospot assay. CTL positivity was defined as when the calculated value (average spot number in the peptide pulse group - average spot number in the negative control group/average spot number in the peptide pulse group × 100) by time was greater than that of day 1, and further when the average spot number in the peptide pulse group was greater than the average spot number and standard deviation range in the negative control group.

Serum concentrations of VEGFR-2 were measured before drug administration on day 1, day 8, and day 29, using Quantikine® Human Soluble VEGFR-2 Immunoassay (R&D Systems, Inc).

### Statistical analysis

Overall survival, 1-year survival and progression-free survival were estimated with the Kaplan-Meier method. To assess differences in overall survival between the elpamotide and historical control groups [5, 6], log-rank tests and the Harrington-Fleming, in which time is weighted and was used in anticipation that the effects of the vaccine would present with time, were used.

Calculation of sample size was based on an additional treatment effect of 15 % in the elpamotide group compared with the 1-year survival rate in the historical control group, which was derived from previous reports [5, 6]. The null hypothesis was “no extension of 1-year survival” to achieve a one-sided type I error of <10 % and a power of >80 %. We estimated that the 1-year survival rate of the historical control group based on patients with BTC was 15–30 %, and expected elpamotide to add a treatment effect of 15 %. When the historical control group was set at 200 patients, the sample size needed for the elpamotide group was calculated to be 45–60 patients. Accordingly, we aimed to select a total of 50 patients.

Serum concentrations of VEGFR-2 were analyzed by post-hoc test. All statistical analyses were conducted with SAS software, version 9.1.3 (SAS Institute).

## Results

### Patient characteristics

Of the 55 patients registered from October 2009 to June 2011, 54 who underwent the trial therapy were included in the full analysis set and safety analysis set. Patient characteristics are summarized in Table 2. Compared to the historical control group, the present trial had higher proportions of patients without gallbladder cancer (66.7 % vs. 45–50.7 %) and those having a performance status of 0 (90.7 % vs. 60 %).

**Table 2:** Patient characteristics (*N* _54)*

Characteristics	No. of patients	%
Age, years		
<65	27	50
≥65	27	50
Sex		
Male	30	55.6
Female	24	44.4
Primary tumour site		
Intrahepatic bile duct	20	37
Gallbladder	18	33.3
Extrahepatic bile duct	13	24.1
Ampulla of vater	3	5.6
Extent of disease		
Metastatic	34	63
Locally advaced	20	37
Resection		
No	37	68.5
Yes	17	31.5
Lymphocyte		
≥18 %	45	83.3
<18 %	9	16.7
PS (ECOG)		
0	49	90.7
1	5	9.3

Clinical characteristics of the 54 patients who received elpamotide+GEM

*PS* (*ECOG*) Performance status (Eastern Cooperative Oncology Group)

### Survival and response rate

Fourteen patients (25.9 %) survived ≥1.5 years, and two completed the 1.5-year trial therapy. The median number of courses of study treatment was 4.5 (range: 1–20), and the dose intensity of elpamotide and Gem was 90.0 and 82.7 %, respectively. Main reasons for discontinuation were exacerbation of primary disease (34 cases) and adverse event-related reasons (6 cases).

**Fig. 1:**
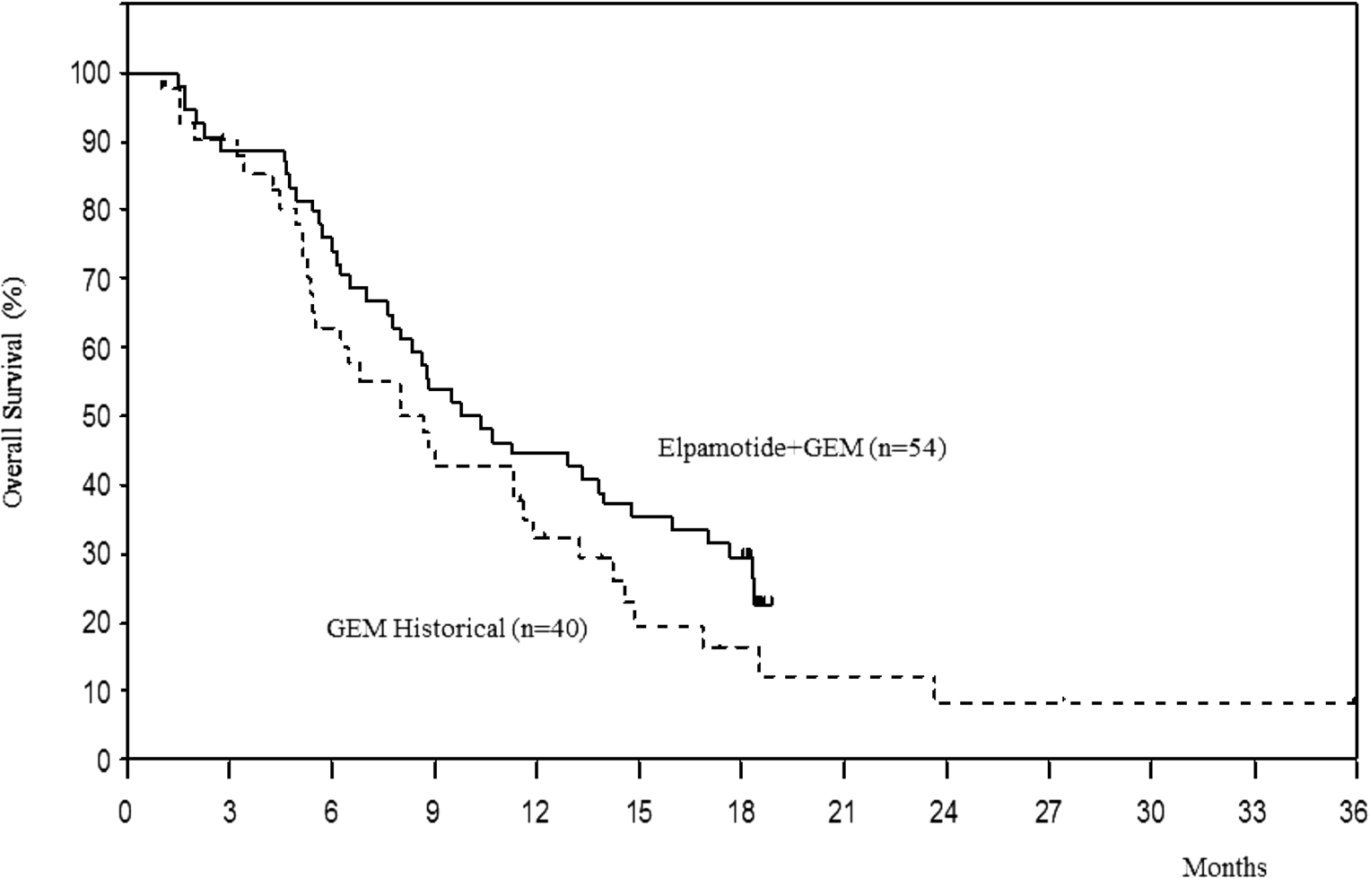
Overall survival

Median survival was 10.1 months (95 % confidence interval (CI): 8.0–14.0 months), which was longer than that of the historical control (7.6 months) (*P* = 0.079; Harrington-Fleming method; *P* = 0.043, log-rank test; Fig. 1). One-year survival rate was 44.4 %, and median progression-free survival was 4.5 months (95 % CI: 2.8–7.1 months).

Median overall survival by site of origin was as follows: intrahepatic bile duct (11.6 months), extrahepatic bile duct (18.3 months), gallbladder (8.4 months), and vater papilla (9.8 months). These were superior to the 8.7, 10.1, 6.5, and 9.3 months, respectively, in the historical control.

None of the patients achieved complete response, while 10 achieved partial response, with the imaging response rate of 18.5 %. Stable disease was maintained for ≥6 months in 8 of 28 patients (14.8 %).

### Toxicity

Major hematologic ADRs included decreased white blood cell counts (75.9 %), decreased platelet counts (72.2 %), and decreased neutrophil counts (64.8 %). Major non-hematologic ADRs included injection site reaction (68.5 %), induration and erythema (64.8 and 27.8 %), nausea (51.9 %), and decreased appetite and malaise (37.0 %). Severe adverse effects were observed in five patients as follows: pneumocystis pneumonia, loss of appetite, thrombotic microangiopathy, interstitial lung disease, and fever. ADRs of grade 3 or higher are summarized in Table 3. There were no treatment-related deaths.

**Table 3:** Adverse drug reactions

Adverse drug reactions	Grade 3		Grade 4	
	*N*	%	*N*	%
Hematological				
Decreased neutrophil count	16	29.6	3	5.6
Decreased lymphocyte count	9	16.7	0	0.0
Decreased white blood cell count	5	9.3	0	0.0
Decreased platelet count	4	7.4	1	1.9
Anemia	2	3.7	0	0.0
Non-hematological				
Pneumocystis jiroveci pneumonia	1	1.9	0	0.0
Thrombotic microangiopathy	1	1.9	0	0.0
Decreased appetite	1	1.9	0	0.0
Interstitial lung disease	1	1.9	0	0.0
Elevated alanine aminotransferase level	1	1.9	0	0.0
Elevated aspartate aminotransferase level	1	1.9	0	0.0
Elevated blood glucose level	1	1.9	0	0.0
Elevated gamma-glutamyltransferase level	1	1.9	0	0.0
Elevated hepatic enzyme level	1	1.9	0	0.0

### Subgroup analysis

**Fig. 2:**
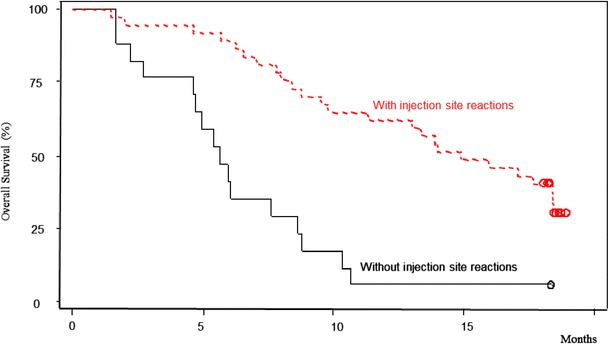
Overall survival with or without injection site reactions

Among 37 patients who developed injection site reactions (ulcer, induration, or erythema), tumor regression was observed in 10 (27 %) during the study period. Moreover, the median overall survival of the 37 patients was significantly longer (14.8 months) compared to that of the remaining 17 who developed no injection site reactions (5.7 months; Table 4 and Fig. 2).

**Table 4:** Relationship between the efficacy and injection site reactions

	With ISR (*n* = 37)	Without ISR (*n* = 17)	
	*N* (%)	*N* (%)	*P*-value
Response			
Complete response (CR)	0 (0.0)	0 (0.0)	
Partial response (PR)	10 (27.0)	0 (0.0)	
Stable disease (SD)	20 (54.1)	8 (47.1)	
Progressive disease (PD)	7 (18.9)	7 (41.2)	
Not evaluable (NE)	0 (0.0)	2 (11.8)	
Overall survival			
Median survival (95 % CI)	14.8 months (9.8, 18.4)	5.7 months (4.6, 8.6)	0.002 (H-F), <0.001 (log-rank)

*CI* confidence interval, *ISR* injection site reaction

### Exploratory analysis

The induction of VEGFR2-specific CTLs was assessed in nine patients; six were positive (66.7 %). There was no clear association between CTL positivity with treatment survival, response rate, or ADRs.

Serum concentrations of VEGFR-2 were evaluated in 43 patients, and found to be significantly increased from baseline (day 1) to day 8 (*P* = 0.015), and significantly decreased from day 8 to day 29 (*P* = 0.010); there was no significant difference from baseline to day 29. Response rate in the 31 patients (72 %) with an elevated serum VEGFR-2 concentration at day 8 was 19 %, and median survival was 13.3 months. There was no apparent association between serum VEGFR-2 concentration and efficacy or ADRs.

## Discussion

Tumor immunotherapy has recently gained much attention, and there are currently more than 100 clinical studies in progress around the world. As a results, some immunotherapeutic drugs already approved [7, 8], and such approval reflects the findings that immunotherapy activates the immune response in cancer patients and is clinically effective.

The present trial was planned and conducted before Gem plus cisplatin therapy became the standard chemotherapy for BTC based on results of the ABC-02 [9] and BT-22 [10] trials. The reliable reference data at the time of planning this trial were only the retrospective data from two studies [5, 6]. Based on results from those studies, we set the threshold 1-year survival rate at 15–30 %, and expected to add a 15 % treatment effect. The result was a 44.4 % 1-year survival rate, which was in line with this prediction. However, the proportion of good performance status cases and of those without gallbladder cancer were high in this trial. Thus, in the comparison with the historical control, improved survival may have been related to patient background, rather than the vaccine’s additive effects. Median survival with the standard Gem plus cisplatin therapy in the ABC-02 and BT22 trials was 11.7 and 11.2 months, respectively. Based on the median survival of 10.1 months in the present trial, single-agent Gem chemotherapy clearly lacks power as a platform for additive effects over elpamotide.

Survival curves for subgroups of patients who did and did not exhibit injection site reactions differed substantially. The fact that those who exhibited injection site reactions showed better long-term results suggests that it can be used as an indicator for early determination of those likely to benefit from therapy. This phenomenon was also observed in the Gem ± elpamotide trial (PEGASUS-PC Study), which targeted advanced pancreatic cancer patients, and although primitive, it may serve as a highly reliable indicator.

In conclusion, combined immunotherapy with Gem and elpamotide was well-tolerated and showed moderate antitumor effects. For future development of therapies, it will be necessary to optimize the target population for which therapeutic effects could be expected.
